# The impact of sequence length and number of sequences on promoter prediction performance

**DOI:** 10.1186/1471-2105-16-S19-S5

**Published:** 2015-12-16

**Authors:** Sávio G Carvalho, Renata Guerra-Sá, Luiz H de C Merschmann

**Affiliations:** 1 Federal University of Ouro Preto, Morro do Cruzeiro, Ouro Preto-MG, Brazil

**Keywords:** promoter prediction, data mining, structural properties

## Abstract

**Background:**

The advent of rapid evolution on sequencing capacity of new genomes has evidenced the need for data analysis automation aiming at speeding up the genomic annotation process and reducing its cost. Given that one important step for functional genomic annotation is the promoter identification, several studies have been taken in order to propose computational approaches to predict promoters. Different classifiers and characteristics of the promoter sequences have been used to deal with this prediction problem. However, several works in literature have addressed the promoter prediction problem using datasets containing sequences of 250 nucleotides or more. As the sequence length defines the amount of dataset attributes, even considering a limited number of properties to characterize the sequences, datasets with a high number of attributes are generated for training classifiers. Once high-dimensional datasets can degrade the classifiers predictive performance or even require an infeasible processing time, predicting promoters by training classifiers from datasets with a reduced number of attributes, it is essential to obtain good predictive performance with low computational cost. To the best of our knowledge, there is no work in literature that verified in a systematic way the relation between the sequences length and the predictive performance of classifiers. Thus, in this work, we have evaluated the impact of sequence length variation and training dataset size (number of sequences) on the predictive performance of classifiers.

**Results:**

We have built sixteen datasets composed of different sized sequences (ranging in length from 12 to 301 nucleotides) and evaluated them using the SVM, Random Forest and *k*-NN classifiers. The best predictive performances reached by SVM and Random Forest remained relatively stable for datasets composed of sequences varying in length from 301 to 41 nucleotides, while *k*-NN achieved its best performance for the dataset composed of 101 nucleotides. We have also analyzed, using sequences composed of only 41 nucleotides, the impact of increasing the number of sequences in a dataset on the predictive performance of the same three classifiers. Datasets containing 14,000, 80,000, 100,000 and 120,000 sequences were built and evaluated. All classifiers achieved better predictive performance for datasets containing 80,000 sequences or more.

**Conclusion:**

The experimental results show that several datasets composed of shorter sequences achieved better predictive performance when compared with datasets composed of longer sequences, and also consumed a significantly shorter processing time. Furthermore, increasing the number of sequences in a dataset proved to be beneficial to the predictive power of classifiers.

## Background

Over recent years, advances in technology have allowed an acceleration of new genomes sequencing [1], evidencing the increasing demand for data analysis automation and for improving procedures previously used [2]. This has encouraged studying and implementing several computational techniques and creating new tools to enable processing of large amounts of genomic data.

One of the first steps for functional genomic annotation is promoter identification. Promoters are regions responsible for signaling and controlling the exact position where the transcription mechanism initiates, called TSS (*Transcription Start Site*). The capability for detecting them in their different forms will make it possible to understand how, where and when transcription occurs, in addition to providing clarification on the interaction network and regulation of the transcription mechanism [3,1].

The identification of promoter sequences in genomes can be seen as a classification problem, where, given the features of a genomic sequence, it would be classified as promoter or non-promoter. Therefore, several computational approaches to predict promoters have been proposed using different classification techniques and different types of information extracted from sequences. Nevertheless, further progress is needed to improve them [4-6,1].

Much of the complexity of promoter prediction problem is due to their diverse nature, which makes it difficult to identify them [7,3,8]. The selection of inappropriate features to predict promoters can result in a high number of false-positives. Therefore, a crucial step for prediction success is to discover features of promoter sequences that are relevant to differentiate them from non-promoter sequences.

In the search for relevant features to distinguish between promoter and non-promoter sequences, several properties of sequences have been tested for their predictive capability. According to [4], a prediction strategy can use three types of features: structural, based on signs and based on context. Several studies have shown that in order to build accurate models to predict or describe genomic processes, the structural properties of the DNA molecules must be considered [9]. Thus, the structural properties have been widely used in recent years [4] and have also been adopted for this work.

Despite the large amount of work involving promoter prediction [7,3,5,2,6,10,1], to the best of our knowledge, none of them have verified in a systematic way the relation between the length of sequences used for training classification models and their predictive performance.

The importance of this evaluation is due to the fact that, considering the structural properties, the longer the sequences used to compose datasets used for training classifiers, the greater the amount of attributes. The problem is that high-dimensional datasets, that is, with great number of attributes, make the classification a more complex process, and the result may be an increase in classifiers training time and a reduction of their predictive performance.

In our preliminary work [11], the effect of sequence length for distinguishing between promoters and nonpromoters was briefly evaluated using two classification techniques. In this work, in order to extend this analysis, an additional classifier, the Random Forest, was evaluated using the same datasets adopted in [11]. Statistical tests were also applied aiming at assessing the differences among the classification performances obtained from each evaluated dataset. Classifiers' performances for each class were included as well. Finally, results of an experiment carried out to evaluate the impact of increasing the number of dataset instances on classifiers predictive accuracy were also added.

Due to the amount of data available and the attention it has received from the scientific community in recent decades [4], the genome chosen to be studied in this work was *Homo sapiens*. The experiments were conducted using a well-known and reliable promoter database which is publicly available on the web.

## Methods

### Consolidation of datasets

For the studies conducted in this work, promoter and non-promoter sequences derived from human genome were used for datasets construction.

Promoters were obtained from a set of sequences available in the DBTSS database [12] (version 8.0), which has already been used in several other works [6,3,1,2], and is a set of approximately 98,000 experimentally validated promoter sequences with active TSS, where each sequence has 1201 bp (base pairs).

Non-promoters correspond to several genomic sequences that were extracted randomly from intergenic regions and from introns and exons [6]. The criteria for obtaining these sequences require that the region is at a minimum distance of 1000 nucleotides from the positions demarcated on CAGE database, indicating transcription regions, and at a minimum distance of 1000 nucleotides from the positions demarcated on RefSeq database, that has informations denoting the beginning and ending positions of genes. Thus, the selection of false non-promoter sequences is avoided. CAGE and RefSeq databases were obtained from *pppBenchmark *tool [13] website http://bioinformatics.psb.ugent.be/webtools/pppbenchmark/.

Due to computational cost to process a sequence dataset, only part of the sequences available at DBTSS database were used for composing datasets for the first part of this study. Thus, a total of 7,000 different promoter sequences were chosen randomly, avoiding the inclusion of noisy sequences. In addition, other 7,000 non-promoter sequences completed the datasets. Therefore, these datasets are composed of the same 14,000 sequences.

However, the length of sequences varies from one dataset to another. For example, the dataset called *250-50 *consists of sequences represented by 301 nucleotides. For promoter sequences, this size is the sum of the number of nucleotides positioned upstream and downstream from TSS (in addition to TSS itself), that is, in the example there are 250 nucleotides upstream and 50 nucleotides downstream from TSS. Therefore, for the same dataset, TSS is always located at the same position in all promoter sequences. Since nonpromoter sequences do not have TSS, their length is simply given by their number of nucleotides. Thus, in *250-50 *dataset, non-promoter sequences are also composed of 301 nucleotides.

In addition to these datasets consisting of 14,000 sequences, which were used to evaluate the impact of sequence length variation on the predictive performance of classifiers, datasets comprised of a higher or greater number of sequences (composed of 41 nucleotides) were built in order to verify the impact of an increased number of training instances on the predictive capacity of classification models. Using the same procedures previously presented for generating datasets, a random selection of 73,770 promoter sequences from the DBTSS database allowed the construction of additional datasets containing 40,000, 50,000 and 60,000 promoter sequences. It is worth noting that the same amount of non-promoter sequences completes each of these datasets, maintaining the ratio of 50% of promoter sequences and 50% of non-promoter sequences.

Each dataset sequence is characterized by a set consisting of 13 structural properties [9], named: A-philicity, base stack energy, B-DNA, bendability, DNA-bending stiffness, disrupt energy, DNA denaturation, free energy, nucleosome positioning, propeller twist, protein deformation, protein-DNA twist and Z-DNA. These properties, which have already been subject of other studies in literature [10,5,1], are physicochemical and conformational properties.

Since the structural properties may be determined by local interactions among neighboring nucleotides in a sequence [9], they are represented by tables where each possible nucleotide combination is associated with a value that represents its contribution to a particular structural property. As an example, Table 1 presents the mapped values of oligonucleotides for the stacking energy structural property.

**Table 1:** Mapped values of oligonucleotides for base stack energy property [14].

**Oligonucl**.	Value (kcal/mole)	**Oligonucl**.	Value (kcal/mole)
AA	-5.37	GA	-9.81
AC	-10.51	GC	-14.59
AG	-6.78	GG	-8.26
AT	-6.57	GT	-10.51
CA	-6.57	TA	-3.82
CC	-8.26	TC	-9.81
CG	-9.69	TG	-6.57
CT	-6.78	TT	-5.37

**Figure 1:**
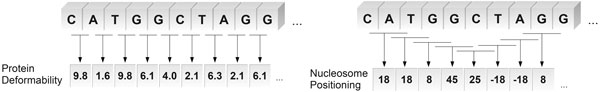
**Conversion of a sequence to two structural properties**.

Using these 13 structural properties, each nucleotide sequence (promoters and non-promoters) is converted into a numerical vector that characterizes it. Figure 1 illustrates the conversion of a sequence into two structural properties (protein deformation and nucleosome positioning). As it can be observed, the numerical vector of each property (structural profile) is obtained from scanning the sequence of nucleotides where, depending on the structural property, each vector value is obtained considering sequences of dinucleotides (protein deformability) or trinucleotides (nucleosome positioning).

**Figure 2:**
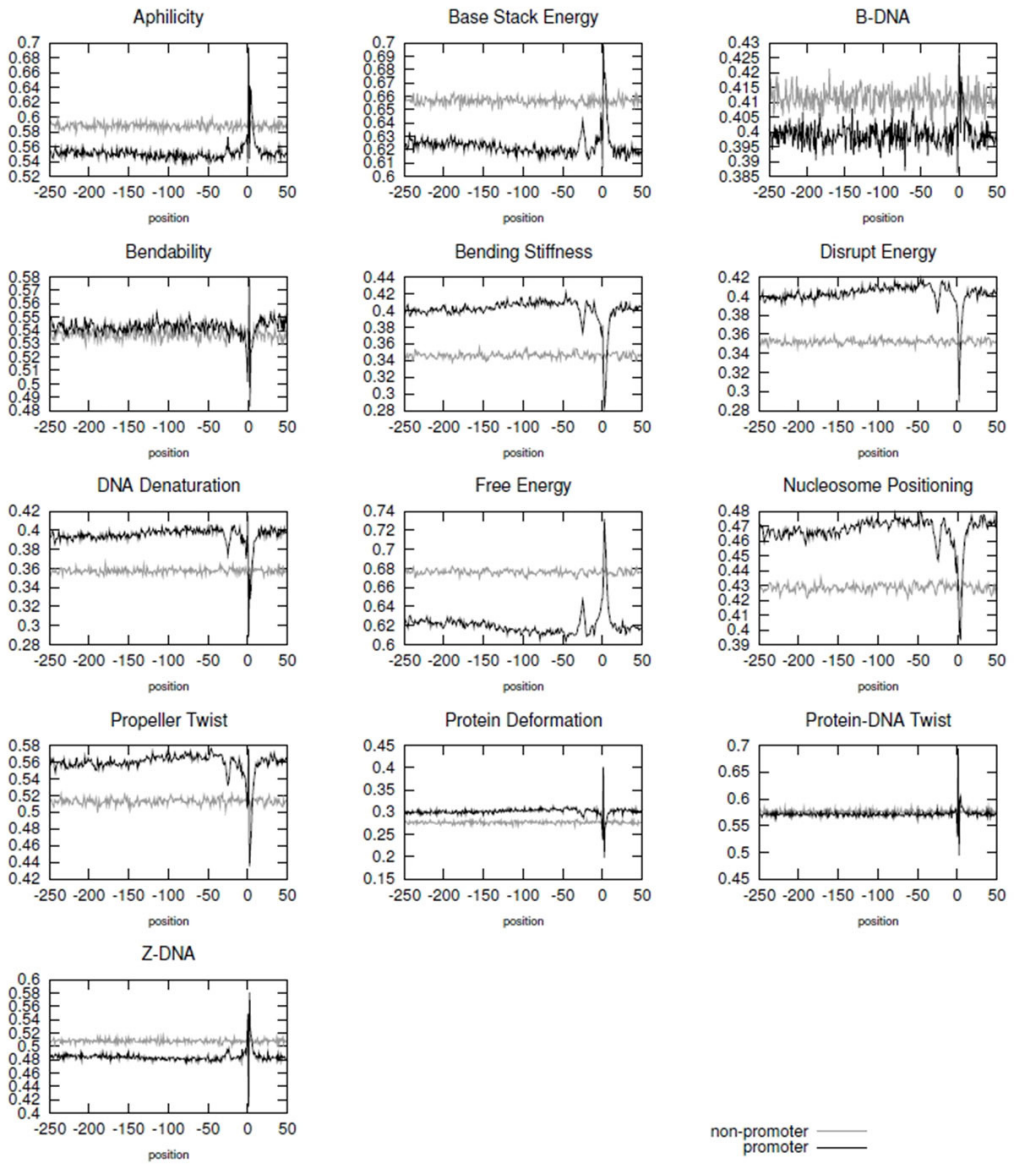
**Average structural profile of thirteen features along promoter and non-promoter sequences of the *250-50 *dataset**. Each graph corresponds to a different structural property, and the property name is presented on the top of the graph.

Considering the conversion schema previously mentioned, in order to show the capability of the structural properties to distinguish promoter from non-promoter sequences, Figure 2 illustrates, for all structural properties considered in this work, the average structural profile of promoter and non-promoter sequences of the *250-50 *dataset. The structural profiles were plotted according to the average value on each position. In this figure, TSS is located at the 0 position.

**Figure 3:**
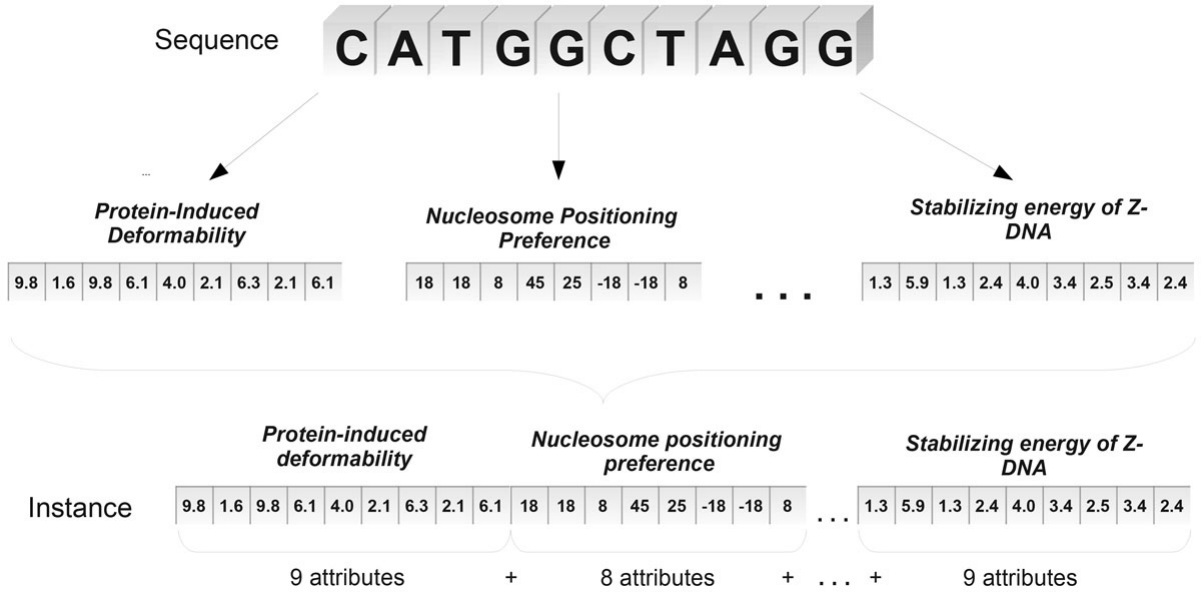
**Process of generating dataset instance from nucleotide sequence**.

The complete characterization of a sequence is given by a single numerical vector resulting from the junction of the vectors representing each of the 13 structural properties considered in this work. The size of the resultant vector of these junctions corresponds to the number of predictor attributes of the datasets used for classifiers training. Figure 3 illustrates this process of generating dataset instances. In addition to these predictor attributes, each sequence has a value for the class attribute, which indicates whether that sequence is promoter or non-promoter. As an example, the largest dataset used in our experiments, the *250-50 *one, results in a set of 3898 predictor attributes. Table 2 shows the number of predictor attributes for each dataset used in this work.

**Table 2:** Number of predictor attributes for each dataset.

Dataset	Number of attributes
10-1	141
10-3	167
10-5	193
10-10	258
10-20	388
10-30	518
10-40	648
10-50	778
20-50	908
30-50	1,038
40-50	1,168
50-50	1,298
100-50	1,948
150-50	2,598
200-50	3,248
250-50	3,898

As it can be observed in Table 2 the length of sequences used to compose the dataset defines the amount of their attributes. Several studies in literature have addressed the problem of promoter prediction using datasets containing sequences of 250 nucleotides or more [7,2,3,1]. Although a limited amount of features is being used in characterization of sequences, high-dimensional datasets are generated for classifiers training.

The problem with high-dimensional datasets, that is, with high number of attributes, is that they make classification a more complex process, often consuming an infeasible time for training classifiers and degrading their predictive performance.

Therefore, to predict promoters by training classifiers from datasets with a reduced number of attributes, it is essential to obtain good predictive performance with low computational cost. This way, the objective of the first set of experiments conducted in this work is to evaluate the impact of the sequence length variation on classifiers performance. After that, considering a limited sequence length (41 nucleotides), additional experiments were carried out aiming at verifying the impact of an increased number of training instances on predictive performance of classifiers.

### Classifiers and experimental setup

SVM (Support Vector Machine) [15], Random Forest [16] and *k*-NN (*k*-Nearest Neighbors) [17] classifiers, usually adopted in data mining works, were chosen to evaluate the impact of the sequence length variation and training dataset size (number of instances) on the performance of predictive models. Experiments were conducted using the caret package (short for *classification and regression training *) in R [18], which is a programming language and an environment widely used in statistical and graphics computation for data analysis.

*k*-NN classifier's idea is very simple. Each dataset instance is described by an *n*-dimensional vector, where *n *corresponds to the number of predictor attributes. To classify a new instance (an instance whose class is unknown), the classifier uses distance metrics to determine the *k *training instances that are more similar to the instance to be classified. Then, the most frequent class among the *k *similar instances is attributed to the new instance. In *k*-NN, the *k *value is an input parameter.

Considering each dataset instance as a point in *n*-dimensional space, the basic idea of SVM is to find a hyperplane with maximum margin of separation, i.e., one that provides the separation of training instances, with maximum margin, in two portions in *n*-dimensional space. Once the optimal hyperplane is found, the classification of a new instance is made by determining its position in relation to the separation hyperplane. Although this method was originally proposed for binary classification problems, several extensions have been proposed in literature to make it suitable for multi-class classification problems.

Random Forest (RF) is a classification method that operates building decision trees and performing classification by combining their results. Through the Bagging process, multiple datasets are derived from the original one and, from each derived dataset, a new decision tree is generated. The construction of these trees is made by using a subset of attributes randomly selected from the original dataset. The random nature of the process ensures low processing cost and diversity of generated decision trees.

In order to set the algorithms parameters for the datasets used in this study, experiments were conducted by varying the parameters values *C *(0.25, 0.5, 1, 2, 4) and *gamma*([0.1, 0.0001]), for SVM (using RBF kernel), *mtry *($\sqrt[{4}]{p}$; $\sqrt{p/2}$; $\sqrt{p}$; $2\sqrt{p}$, *p*/2), where *p *is the number of predictive attributes of the dataset, for Random Forest (using *ntree *= 500) and *k *(1, 3, 5, 7, 9) for *k*-NN. Table 3 presents the best parameter values obtained for each dataset and therefore used in our experiments to obtain the results presented here. All experiments were carried out on a Core i7-2600 @ 3.40GHz PC with 12 GBytes of RAM.

**Table 3:** *k*-NN, RF and SVM parameters.

	*k*-NN	RF		SVM	
	
Datasets	*k*	*ntree*	*mtry*	*C*	*sigma*
10-1	9	500	3.45	1	3.64e-03
10-3	9	500	3.59	0.5	3.05e-03
10-5	9	500	6.95	0.5	1e-02
10-10	9	500	8.03	2	1e-03
10-20	9	500	19.7	1	1e-03
10-30	9	500	4.77	1	1e-03
10-40	9	500	25.5	1	7.84e-04
10-50	9	500	55.8	1	1e-03
20-50	9	500	30.1	1	1e-03
30-50	9	500	64.4	0.5	1e-03
40-50	9	500	17.1	0.5	1e-03
50-50	7	500	72.1	0.5	1e-03
100-50	9	500	88.3	1	1e-03
150-50	9	500	102	0.5	1.96e-04
200-50	9	500	114	1	1.56e-04
250-50	9	500	125	1	1e-04

The classifiers predictive performance was measured using ten-fold cross validation method [19] and Fmeasure metric. Furthermore, an agreement statistic, named kappa measure, was also adopted aiming at evaluating the classifiers for datasets composed of different number of sequences. Since this measure do not take into account the correct classification as a result of a mere coincidental concordance between the classifier output and the actual class of each instance to be classified, it is a reliable metric for assessing the performance of classifiers. For each dataset, the same test partitions were used in the evaluation of classifiers.

## Results and discussion

### Impact of the variation of sequences length

**Figure 4:**
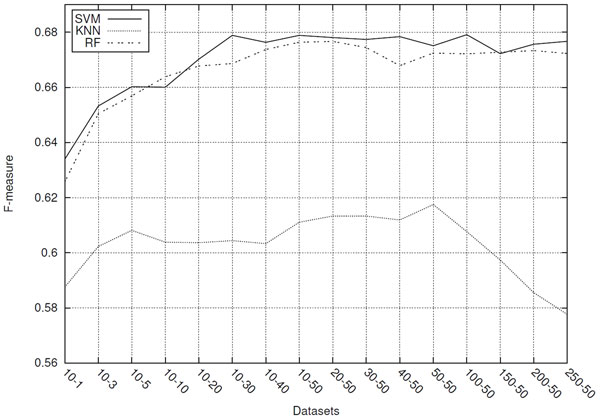
**Classifiers performances for datasets composed of different sized sequences**.

The results obtained from the experiments to verify the impact of the sequence length variation on the classifiers performance are shown in the Figure 4.

The graph of Figure 4 shows the predictive performance, in terms of average *F-measure*, of the SVM, RF and *k*-NN classifiers for each of the 16 evaluated datasets. As it can be seen in this graph, the SVM and RF classifiers have obtained better predictive performance than the *k*-NN one for all datasets evaluated.

Yet, the most important thing to observe in Figure 4 graph is that, for all classifiers, the decrease in the length of sequences used in the datasets did not necessarily imply a reduction in their predictive performance. SVM and RF performance remained relatively stable for datasets composed of sequences ranging in length from 301 (*250-50 *) to 41 (*10-30 *) nucleotides, presenting some degradation in performance only for sequences containing less than 41 nucleotides. *k*-NN achieved its best performance with the *50-50 *dataset and, even for the dataset composed of shorter sequences (*10-1*), presented superior predictive performance compared with larger datasets (*250-50*).

In order to determine if there is a statistically significant difference among the F-measures of the evaluated datasets for each classifier, we have used Friedman test [20]. After that, if the differences in the datasets' performances were statistically significant, the Nemenyi post-hoc test [20] was applied to find which datasets actually differ. These statistical tests were applied with 95% of confidence level.

Table 4 shows the p-value obtained by Friedman test for each classifier evaluated in this study. As all p-values are smaller than 0.05, we can conclude that there is a significant difference among the 16 datasets for all classifiers.

**Table 4:** P-value obtained from the Friedman Test.

Classifier	*P-value*
SVM	1.622e-11
RF	5.556e-09
*K*NN	1.263e-08

Aiming at verifying in which pairs of datasets the differences in performance were statistically significant, the Nemenyi post-hoc test was applied and its results are shown in Tables 5, 6 and 7 for SVM, RF and *k*-NN, respectively. In these tables, the result contained in each intersection of a row and a column indicates if the performances of datasets related to this row and to this column are significantly different (coded as T - true) or not (coded as F - false).

**Table 5:** Nemenyi post-hoc test results for the SVM classifier.

	10-1	10-3	10-5	10-10	10-20	10-30	10-40	10-50	20-50	30-50	40-50	50-50	100-50	150-50	200-50	250-50
10-1	F	-	-	-	-	-	-	-	-	-	-	-	-	-	-	-
10-3	F	F	-	-	-	-	-	-	-	-	-	-	-	-	-	-
10-5	F	F	F	-	-	-	-	-	-	-	-	-	-	-	-	-
10-10	F	F	F	F	-	-	-	-	-	-	-	-	-	-	-	-
10-20	F	F	F	F	F	-	-	-	-	-	-	-	-	-	-	-
10-30	F	F	F	F	F	F	-	-	-	-	-	-	-	-	-	-
10-40	**T**	F	F	F	F	F	F	-	-	-	-	-	-	-	-	-
10-50	**T**	**T**	**T**	F	F	F	F	F	-	-	-	-	-	-	-	-
20-50	**T**	**T**	F	F	F	F	F	F	F	-	-	-	-	-	-	-
30-50	**T**	F	F	F	F	F	F	F	F	F	-	-	-	-	-	-
40-50	F	F	F	F	F	F	F	F	F	F	F	-	-	-	-	-
50-50	**T**	F	F	F	F	F	F	F	F	F	F	F	-	-	-	-
100-50	**T**	F	F	F	F	F	F	F	F	F	F	F	F	-	-	-
150-50	**T**	F	F	F	F	F	F	F	F	F	F	F	F	F	-	-
200-50	**T**	F	F	F	F	F	F	F	F	F	F	F	F	F	F	-
250-50	**T**	F	F	F	F	F	F	F	F	F	F	F	F	F	F	F

**Table 6:** Nemenyi post-hoc test results for the RF classifier.

	10-1	10-3	10-5	10-10	10-20	10-30	10-40	10-50	20-50	30-50	40-50	50-50	100-50	150-50	200-50	250-50
10-1	F	-	-	-	-	-	-	-	-	-	-	-	-	-	-	
10-3	F	F	-	-	-	-	-	-	-	-	-	-	-	-	-	
10-5	F	F	F	-	-	-	-	-	-	-	-	-	-	-	-	
10-10	F	F	F	F	-	-	-	-	-	-	-	-	-	-	-	
10-20	F	F	F	F	F	-	-	-	-	-	-	-	-	-	-	
10-30	F	F	F	F	F	F	-	-	-	-	-	-	-	-	-	
10-40	**T**	F	F	F	F	F	F	-	-	-	-	-	-	-	-	
10-50	**T**	**T**	**T**	F	F	F	F	F	-	-	-	-	-	-	-	
20-50	**T**	**T**	F	F	F	F	F	F	F	-	-	-	-	-	-	
30-50	**T**	F	F	F	F	F	F	F	F	F	-	-	-	-	-	
40-50	F	F	F	F	F	F	F	F	F	F	F	-	-	-	-	
50-50	**T**	F	F	F	F	F	F	F	F	F	F	F	-	-	-	
100-50	**T**	F	F	F	F	F	F	F	F	F	F	F	F	-	-	-
150-50	**T**	F	F	F	F	F	F	F	F	F	F	F	F	F	-	-
200-50	**T**	F	F	F	F	F	F	F	F	F	F	F	F	F	F	-
250-50	**T**	F	F	F	F	F	F	F	F	F	F	F	F	F	F	F

**Table 7:** Nemenyi post-hoc test results for the kNN classifier.

	10-1	10-3	10-5	10-10	10-20	10-30	10-40	10-50	20-50	30-50	40-50	50-50	100-50	150-50	200-50	250-50
10-1	F	-	-	-	-	-	-	-	-	-	-	-	-	-	-	-
10-3	F	F	-	-	-	-	-	-	-	-	-	-	-	-	-	-
10-5	F	F	F	-	-	-	-	-	-	-	-	-	-	-	-	-
10-10	F	F	F	F	-	-	-	-	-	-	-	-	-	-	-	-
10-20	F	F	F	F	F	-	-	-	-	-	-	-	-	-	-	-
10-30	F	F	F	F	F	F	-	-	-	-	-	-	-	-	-	-
10-40	F	F	F	F	F	F	F	-	-	-	-	-	-	-	-	-
10-50	**T**	F	F	F	F	F	F	F	-	-	-	-	-	-	-	-
20-50	**T**	F	F	F	F	F	F	F	F	-	-	-	-	-	-	-
30-50	**T**	F	F	F	F	F	F	F	F	F	-	-	-	-	-	-
40-50	**T**	F	F	F	F	F	F	F	F	F	F	-	-	-	-	-
50-50	**T**	F	F	F	F	F	F	F	F	F	F	F	-	-	-	-
100-50	F	F	F	F	F	F	F	F	F	F	F	F	F	-	-	-
150-50	F	F	F	F	F	F	F	F	F	F	F	F	F	F	-	-
200-50	F	F	F	F	F	F	F	F	**T**	**T**	F	**T**	F	F	F	-
250-50	F	F	**T**	F	F	F	F	**T**	**T**	**T**	**T**	**T**	**T**	F	F	F

For the SVM classifier, based on the results shown in Table 5 it can be noted that the dataset, composed of smaller sequences, that does not present a statistically significant difference in classification performance when compared with any other datasets comprising longer sequences, is the *10-20*.

The results presented in Table 6 show that, for RF classifier, the classification performance of the dataset composed of sequences of 21 nucleotides (*10-10*) is not statistically inferior to any dataset comprising sequences longer than 21 nucleotides.

Similarly to what happened to the SVM and RF classifiers, the results displayed in Table 7 for *k*-NN show a dataset composed of short sequences (14 nucleotides) that achieved no worse classification performance (with statistical significance) than those obtained by datasets composed of longer sequences.

The results presented so far show that, for the evaluated classifiers, datasets consisting of sequences represented by 31 or less nucleotides allow the construction of classification models that are as accurate as those obtained from datasets composed of longer sequences, commonly adopted in literature for promoter prediction purpose.

The importance of this result is due to the fact that the size of the sequences used for training classifiers can make the process very complex, often degrading their predictive performance or consuming unfeasible processing time and computational resources.

**Figure 5:**
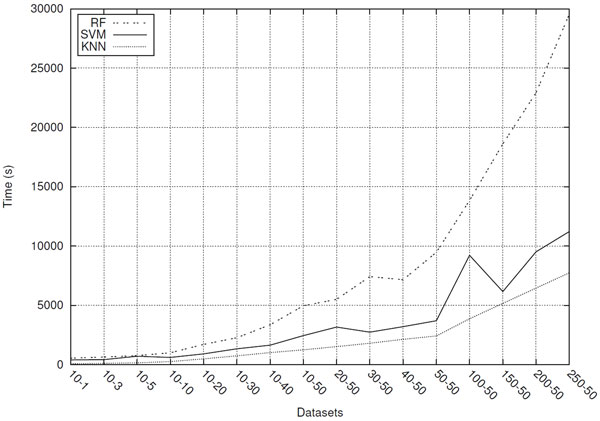
**Classification processing times for datasets composed of different sized sequences**.

Figure 5 graph shows that, for the three evaluated classifiers, the time spent for processing datasets grows exponentially as the lengths of their sequences increase. It is worth noting that, in many cases, a dataset composed of shorter sequences achieves superior predictive performance and consumes much less processing time than datasets composed of longer sequences. For example, for SVM, the *10-30 *dataset presents a slightly higher predictive performance than that achieved by the *250-50 *dataset (see Table 5) and the processing time is more than 8 times shorter than that spent by the *250-50 *dataset. Even greater is the difference presented by Random Forest method, in which the time spent for processing the *10-30 *dataset is about 12 times shorter than that spent by *250-50 *dataset.

**Figure 6:**
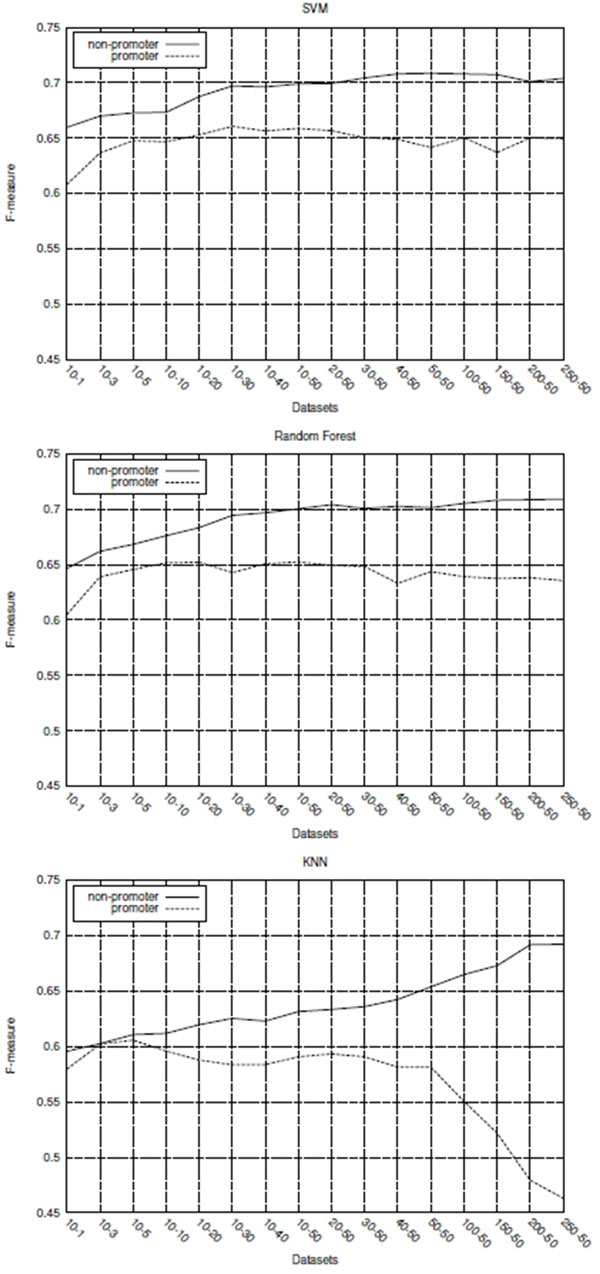
**Average F-measure by class for datasets composed of different sized sequences**.

The average F-measure results presented so far have been computed considering test instances of both classes, promoter and non-promoter. Although all datasets used in the experiments are balanced in the class distribution, classifiers performances for each class are not necessarily similar. Then, the graphs in Figure 6 represent the *F-measure *value of each class for all three classifiers and for the 16 evaluated datasets. These graphs show that all classifiers had a better predictive performance for non-promoter class for all datasets. It is also worth noting that, for all classifiers, the performance difference between the classes is always smaller for datasets composed of shorter sequences. This outcome reinforces the importance of training classifiers from datasets consisting of sequences represented by fewer nucleotides.

### Impact of increasing the number of instances

As previously mentioned, due to the computational cost for processing a dataset of sequences, only part of the promoter sequences obtained from the DBTSS database was considered for composing datasets used in the experiments involving the evaluation of the length variation on predictive performance of classifiers. From these experiments, it was found that datasets consisting of sequences represented by few nucleotides (less than 50) resulted in predictive models equivalent to those obtained from datasets formed by longer sequences.

Since the length reduction of sequences used in datasets can substantially reduce the processing time (see Figure 5) and the amount of memory consumed in classification process, using sequences represented by only 41 nucleotides (in the pattern *10-30 *), additional experiments were conducted in order to answer the following question: could the predictive performance of classifiers be improved by increasing the number of instances (sequences) in datasets?

**Figure 7:**
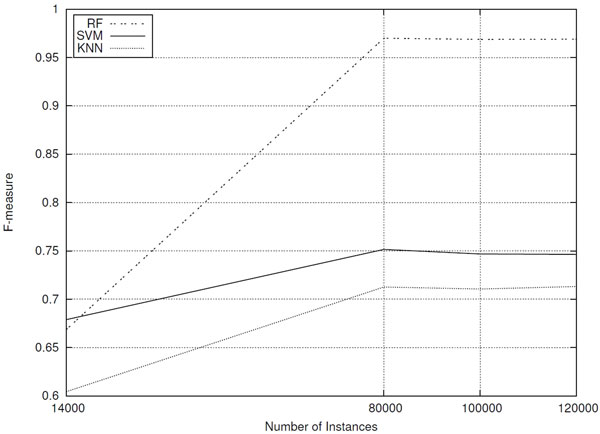
**Classifiers performances (F-measure) for datasets composed of different number of sequences**.

So that we could answer the previous question, datasets containing 80,000, 100,000 and 120,000 instances were built and evaluated. Each dataset was composed by 50% of promoter sequences and 50% of non-promoter sequences. The averages *F-measure *reached by the same classifiers adopted in the previous experiments are shown in Figure 7 graph. In addition, for each classifier, the kappa statistic is presented in order to show its relative improvement over a random predictor.

Figure 7 graph shows that increasing the number of instances for training classifiers can improve their predictive power. All classifiers achieved better predictive performance after increasing the number of instances in the dataset. Random Forest classifier excels in terms of predictive accuracy, achieving F-measure equal to 97% for datasets containing 80,000 instances or more. Furthermore, it is worth noting that, for all classifiers, increasing beyond 80,000 instances does not improve their predictive capacity. This result indicates that using 80,000 sequences is enough to train classification models with good generalization capacity.

**Figure 8:**
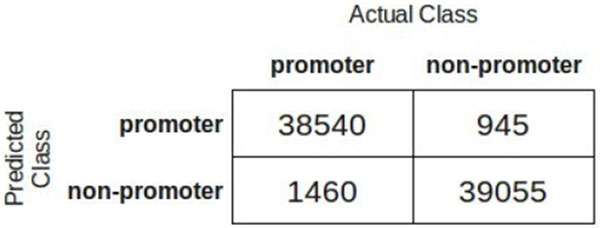
**Random Forest confusion matrix for the dataset containing 80,000 sequences**.

In order to permit a more detailed examination of results reached by RF for the dataset containing 80,000 sequences, its confusion matrix is shown in Figure 8.

**Figure 9:**
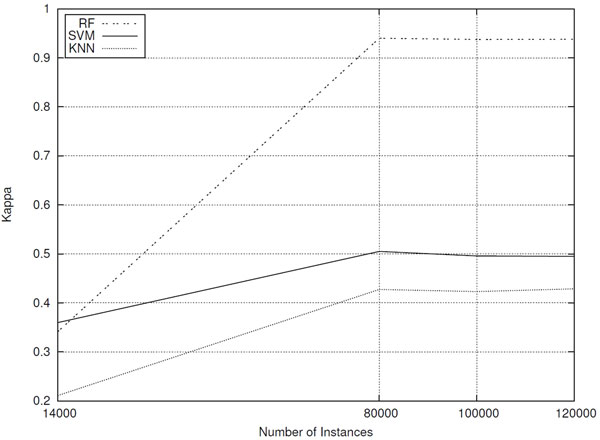
**Classifiers performances (kappa statistic) for datasets composed of different number of sequences**.

In a similar way, kappa statistic improved for all classifiers after increasing the number of instances in the dataset (see Figure 9 graph). The kappa statistic measures the agreement of prediction with the actual class, assuming its maximum value of 1 only when there is a complete agreement. There is not a standardized interpretation of the kappa statistic, but according to [21], values of kappa from 0 to 0.2 are considered slight, 0.21 to 0.4 fair, 0.41 to 0.6 moderate,

1.61 to 0.8 substantial, and 0.81 to 1 as almost perfect. Hence, in spite of the results are regarded as fair for all evaluated classifiers considering the dataset containing 14,000 instances, from 80,000 instances, while *k*-NN and SVM predictive performances are considered moderate, the RF classifier achieved an almost perfect agreement (*kappa *= 0.94).

**Figure 10:**
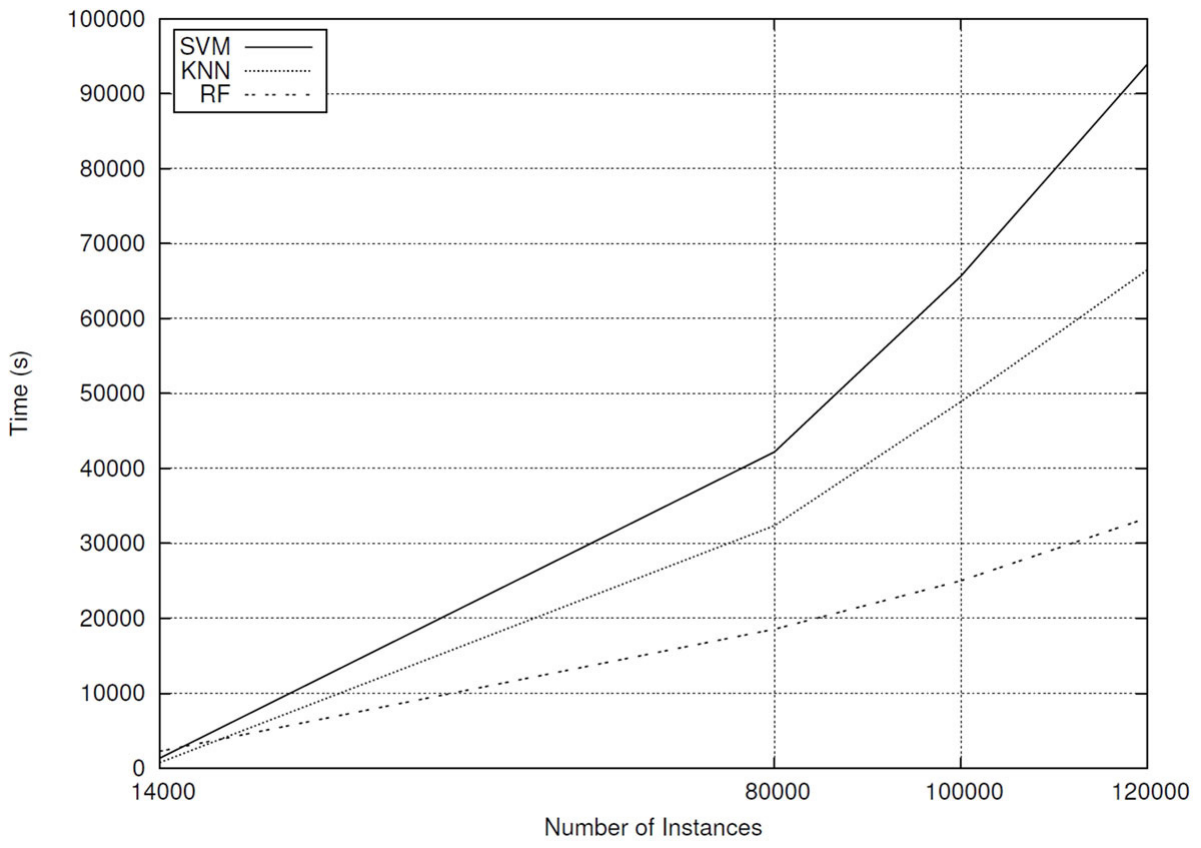
**Classification processing times for datasets composed of different number of sequences**.

Regarding the time spent for processing the datasets, Figure 10 graph shows that it grows roughly linearly as the number of instances in the dataset increases. Another relevant fact is that the RF, classifier that achieves the best predictive performance, is not the one that consumes more processing time.

## Conclusion

Promoter prediction is a fundamental step for genome functional annotation and, therefore, several computational approaches have been proposed using different classification techniques. However, to the best of our knowledge, none of them verified in a systematic way the relation between the length of sequences used for training classification models and their predictive performance. This way, experiments were conducted to analyze the impact of the sequence length variation on the classifiers performance.

In order to perform the analysis previously mentioned, 16 datasets composed of different sized sequences were generated and evaluated using the SVM, RF and *k*-NN classifiers. The experimental results show that a decrease in the length of sequences used in the composition of the datasets did not necessarily result in a reduction of classifiers predictive performance. In addition, several datasets composed of shorter sequences achieved superior predictive performance compared with datasets composed of longer sequences and consumed a significantly shorter processing time. The results show that sequences represented by fewer nucleotides (less than 50) are good enough for human promoters prediction.

From the conclusion obtained in this first experiment, using sequences composed of only 41 nucleotides, datasets with many more instances could be processed to generate new classification models. This way, it was possible to evaluate if larger sets of instances for training classifiers could provide an improvement in their predictive performances. The results have shown that all classifiers achieved better predictive performance after increasing the number of instances in the dataset. The highlight result of this experiment was the RF performance for datasets containing 80,000 sequences or more, which reached F-measure equal to 97%.

Aiming at confirming the predictive power of classifiers, the kappa measure was also considered in the experimental evaluation. Again, for datasets containing from 80,000 sequences composed of 41 nucleotides, according to interpretation of the kappa statistic presented in [21], while *k*-NN and SVM achieved a moderate predictive performance, RF reached an outstanding performance (*kappa *= 0.94).

As future work, we plan to apply techniques for selecting attributes in datasets generated in this study aiming at reducing the datasets number of attributes and improving classifiers predictive performance.

## Competing interests

The authors declare that they have no competing interests.

## Authors' contributions

SGC, RGS and LHCM conceived the study and designed the experiments. SGC performed the experiments. SGC and LHCM analysed the data and wrote the manuscript. All authors read and approved the manuscript.
